# Supplementary material for: Impact of Ocean Acidification on the Gut Histopathology and Intestinal Microflora of Exopalaemon carinicauda
Source: Animals (Basel). 2023 Oct 23;13(20):3299. doi: 10.3390/ani13203299 (PMC10603730; doi:10.3390/ani13203299)
Supplement: Supplementary file 1 [file animals-13-03299-s001.zip › animals-2642524-supplementary.pdf]

Supplementary Material

Impact of ocean acidification on the gut histopathology and intestinal microflora of *Exopalaemon carinicauda*

Table S1: Sequencing results of different samples.

Sample	RawPE	Combined	Qualified	Nochime	Base(nt)	Avglen(nt)	GC	Q20	Q30
CONT 1	128722	117480	115007	108659	45328305	417.16	52.12%	97.11%	91.21%
CONT 2	116631	104027	101312	86412	36597758	423.53	52.44%	96.08%	89.05%
CONT 3	120815	108940	106520	89172	37532657	420.90	52.52%	96.58%	90.05%
CONT 4	130199	126525	123730	120899	49139609	406.45	53.58%	98.22%	94.18%
CONT 5	123087	109827	107158	95694	40283731	420.96	52.94%	96.74%	90.41%
CONT 6	128486	115174	111838	104209	44336096	425.45	50.98%	96.23%	89.50%
AC74 1	126051	112353	109545	98034	41113585	419.38	53.00%	96.68%	90.34%
AC74 2	128229	116329	113653	92914	38489246	414.25	52.98%	96.87%	90.64%
AC74 3	130822	117451	114852	98041	41011902	418.31	52.43%	96.98%	90.92%
AC74 4	124685	110650	107854	100047	42147755	421.28	52.55%	96.43%	89.84%
AC74 5	128518	112316	109584	97639	41212080	422.09	52.90%	96.55%	89.99%
AC74 6	86732	78571	76592	70197	29404904	418.89	52.90%	97.02%	91.11%
AC70 1	127867	115067	112521	98933	41301690	417.47	53.25%	96.91%	90.75%
AC70 2	124632	112403	109743	96692	40498228	418.84	52.85%	96.81%	90.69%

AC70 3	126236	114266	111738	104513	44388608	424.72	52.33%	96.88%	90.71%
AC70 4	115955	104521	102192	94685	39718497	419.48	52.60%	96.98%	91.07%
AC70 5	61395	55297	54040	49013	20500355	418.26	53.75%	97.11%	91.39%

Notes on Supplementary Table S1.

RawPE: remove the sequence of barcode and primer; Combined: a spliced sequence; Qualified: the sequence after quality control is completed; Nochime: sequence after chimera removal.

Supplemental figure

Figure S1. Survival rate of *Exopalaemon carinicauda* in control group (pH8.1) and experimental group (pH7.4 and 7.0).

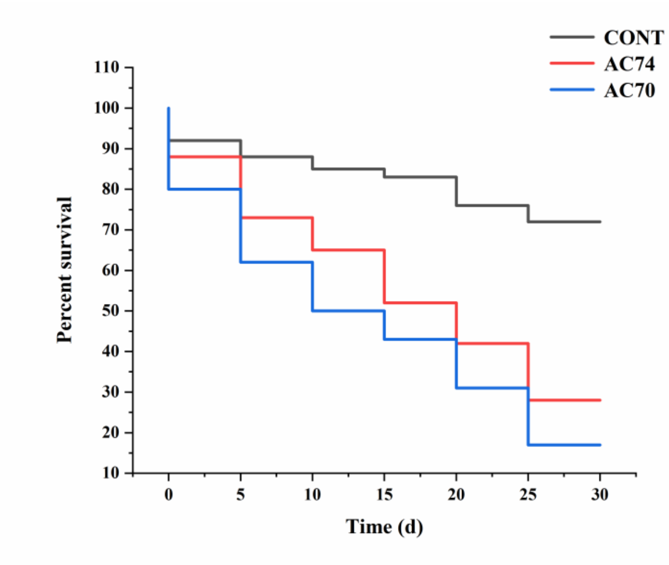

Figure S1. The vertical ladder diagram shows the survival rates of three groups (CONT, AC74 and AC70).
